# Epidemiological Insights into the Omicron Outbreak via MeltArray-Assisted Real-Time Tracking of SARS-CoV-2 Variants

**DOI:** 10.3390/v15122397

**Published:** 2023-12-08

**Authors:** Ting Yan, Rongrong Zheng, Yinghui Li, Siyang Sun, Xiaohong Zeng, Zhijiao Yue, Yiqun Liao, Qinghua Hu, Ye Xu, Qingge Li

**Affiliations:** 1 Engineering Research Centre of Molecular Diagnostics of the Ministry of Education, State Key Laboratory of Cellular Stress Biology, State Key Laboratory of Molecular Vaccinology and Molecular Diagnostics, School of Life Sciences, Faculty of Medicine and Life Sciences, Xiamen University, Xiamen 361102, China; tingyan@stu.xmu.edu.cn (T.Y.); siysun@stu.xmu.edu.cn (S.S.); yqliao@xmu.edu.cn (Y.L.); 2 Xiamen Centre for Disease Control and Prevention, Xiamen 361021, China; rong3018@163.com (R.Z.); zengxiaohong2008@126.com (X.Z.); 3 Shenzhen Centre for Disease Control and Prevention, Shenzhen 518055, China; liyh@szcdc.net (Y.L.); zhijy2823@163.com (Z.Y.); huqinghua03@163.com (Q.H.)

**Keywords:** COVID-19, omicron subvariants, multiplex PCR, wastewater surveillance, SARS-CoV-2

## Abstract

The prolonged course of the COVID-19 pandemic necessitates sustained surveillance of emerging variants. This study aimed to develop a multiplex real-time polymerase chain reaction (rt-PCR) suitable for the real-time tracking of Omicron subvariants in clinical and wastewater samples. Plasmids containing variant-specific mutations were used to develop a MeltArray assay. After a comprehensive evaluation of both analytical and clinical performance, the established assay was used to detect Omicron variants in clinical and wastewater samples, and the results were compared with those of next-generation sequencing (NGS) and droplet digital PCR (ddPCR). The MeltArray assay identified 14 variant-specific mutations, enabling the detection of five Omicron sublineages (BA.2*, BA.5.2*, BA.2.75*, BQ.1*, and XBB.1*) and eight subvariants (BF.7, BN.1, BR.2, BQ.1.1, XBB.1.5, XBB.1.16, XBB.1.9, and BA.4.6). The limit of detection (LOD) of the assay was 50 copies/reaction, and no cross-reactivity was observed with 15 other respiratory viruses. Using NGS as the reference method, the clinical evaluation of 232 swab samples exhibited a clinical sensitivity of > 95.12% (95% CI 89.77–97.75%) and a specificity of > 95.21% (95% CI, 91.15–97.46%). When used to evaluate the Omicron outbreak from late 2022 to early 2023, the MeltArray assay performed on 1408 samples revealed that the epidemic was driven by BA.5.2* (883, 62.71%) and BF.7 (525, 37.29%). Additionally, the MeltArray assay demonstrated potential for estimating variant abundance in wastewater samples. The MeltArray assay is a rapid and scalable method for identifying SARS-CoV-2 variants. Integrating this approach with NGS and ddPCR will improve variant surveillance capabilities and ensure preparedness for future variants.

## 1. Introduction

The coronavirus disease (COVID-19) pandemic has persisted for over three years and remains a global concern. Although the World Health Organization (WHO) declared that COVID-19 is no longer a public health emergency of international concern (PHEIC) on 5 May 2023, continuous surveillance remains pivotal for vaccine improvement and effective management because of the evolution of the severe acute respiratory syndrome coronavirus 2 (SARS-CoV-2). The SARS-CoV-2 Omicron variant has subsequently diverged into numerous sublineages, such as BA.2*, BA.5.2*, BA.2.75*, BQ.1* (BA.5.3.1.1.1.1.1), and XBB.1* [1]. These sublineages, along with their subvariants [BF.7 (BA.5.2.1.7), BN.1 (BA.2.75.5.1), BR.2 (BA.2.75.4.2), BQ.1.1 (BA.5.3.1.1.1.1.1.1), XBB.1.5, XBB.1.16, XBB.1.9, and BA.4.6], exhibit increased transmissibility and immune evasion [2,3], potentially exacerbating the pandemic.

On 7 December 2022, China experienced an Omicron outbreak following adjustments to its prevention and control policies [4]. The surge in COVID-19 cases has raised concerns regarding whether it was driven by emerging SARS-CoV-2 variants [5]. To gain a comprehensive understanding of the evolution of the outbreak in China after lifting COVID-19 restrictions, real-time and continuous variant surveillance is imperative.

The identification of SARS-CoV-2 variants primarily relies on next-generation sequencing (NGS) [6], however, it is time-consuming, expertise-demanding, labor-intensive, and challenging to perform in real time [7]. As a complementary approach, real-time polymerase chain reaction (rtPCR) has been widely employed for SARS-CoV-2 surveillance due to its convenience and accessibility [8,9]. Nevertheless, conventional rtPCR-based methods have limitations in terms of their multiplexing capacity owing to methodological constraints. In this study, we leveraged MeltArray, a highly multiplex PCR approach that can detect 10-fold more targets than conventional rtPCR in one reaction [10], to incorporate the latest variant-specific mutations for the rapid identification of the prevalent Omicron sublineages and subvariants. The established MeltArray assay was used to gain epidemiological insights into the Omicron outbreak via real-time tracking of Omicron variants in clinical and wastewater samples.

## 2. Methods

### 2.1. Plasmids and Virus Strains

Plasmids containing either wild-type or SARS-CoV-2 variant-specific mutations used for assay development were synthesized by Amoy Nucleotide Biotechnology (Xiamen, China). Their original concentrations were determined using an ND-1000 spectrophotometer (NanoDrop Technologies, Wilmington, DE, USA). Cultured viruses, including BF.7, BQ.1, BN.1, XBB.1.5, XBB.1.16, and XBB.1.9 were used as reference strains. Those isolated viruses were cultured using vero cell line. Their sequences were determined via whole-genome sequencing (WGS). Their concentrations were determined via droplet digital PCR (ddPCR) of the N gene (Supplementary File S1). Fifteen other respiratory virus strains used for specificity assessment were provided by Hexin Biotechnology (Guangzhou, China): human coronavirus NL63, human coronavirus 229E, human coronavirus OC43, human rhinovirus 52, human parainfluenza virus 1, human parainfluenza virus 3, influenza virus A (H3N2), influenza virus B/Yamagata, influenza virus B/Victoria, human respiratory syncytial virus A, human respiratory syncytial virus B, human metapneumovirus A2, human bocavirus, human adenovirus type 3, and mycoplasma pneumoniae.

### 2.2. Sample Collection and Processing

This study was approved by the Medical Ethics Committee of Xiamen University School of Medicine (XDYX2021016, 01/07/2021). Three batches of samples were used in this clinical study. The first batch comprised 239 SARS-CoV-2 RNA-positive nasopharyngeal swabs collected at the Xiamen Centre for Disease Control and Prevention between October 2022 and January 2023, which corresponded to the Omicron outbreak after the policy change. The 239 samples had two channels: 95 samples were collected from patients in Xiamen (local), and 144 were collected from international travelers arriving in Xiamen (imported). The second batch comprised 1408 SARS-CoV-2 RNA positive oropharyngeal swabs collected in Xiamen by Zeesan Medical Laboratory between 16 December 2022 and 16 January 2023. The third batch comprised 88 SARS-CoV-2 RNA-positive wastewater samples collected in Shenzhen during the second outbreak of Omicron between 8 May 2023 and 8 June 2023. The virus in the wastewater samples was enriched via a modified PEG precipitation method [11]. Detailed procedures for RNA extraction and real-time reverse transcription (RT)-PCR of the three sample batches are described in the Supplementary File S2.

### 2.3. MeltArray Assay

Sample cDNA was obtained using the LF03 reverse transcription kit (Zeesan Biotech, Xiamen, China), according to the manufacturer’s instructions. The 15-target MeltArray assay for Omicron subvariant identification contained eight pairs of primers, fourteen mediator probes, a TaqMan probe (Supplementary Table S1), and nine universal molecular beacon reporters (Zeesan Biotech). The MeltArray assay was performed in a 25-μL solution containing PCR master buffer, 8 mM MgCl_2_, 0.4 mM dNTPs, 2 U Taq DNA polymerase (Zeesan Biotech), and 5 μL of cDNA template. PCR and melting curve analysis were performed using the SLAN 96S real-time PCR instrument as follows: denaturation at 95 °C for 2 min; 50 cycles of 95 °C for 20 s, 60 °C for 1 min; 35 °C for 40 min, 95 °C for 2 min, 40 °C for 2 min, followed by an increase from 40 °C to 95 °C (0.16 °C/s). The fluorescence intensity was measured in four detection channels (Atto 425, FAM, HEX, ROX, Cy5, and Quasar 705) at each step of the continuous temperature increase during the melting curve analysis.

### 2.4. Analytical Evaluation of MeltArray Assay

To determine the limit of detection (LOD), a series of 10-fold diluted templates ranging from 1 to 10^4^ copies/μL were detected 20 times at each concentration. Positive results were determined based on the presence of an N gene amplification profile and variant-specific melting curves. The lowest concentration with a positive detection rate of 95% for all targets was considered the LOD, with its matched quantification cycle (Cq) taken as the threshold for result interpretation. The reproducibility of the assay was determined by performing 24 replicate experiments in three batches (at a concentration of 10^2^ copies/μL), from which the three-fold standard deviation (3 SD) and coefficient of variation (CV) for each average *T_m_* value and Cq were calculated. Note that the result for N658S was acquired from a plasmid because of the lack of BA.4.6 sample. The analytical specificity of the assay was assessed via detecting nucleic acids (at a concentration of 10^7^ copies/mL) extracted from the fifteen other respiratory viruses listed above.

### 2.5. NGS

WGS for the first batch of 239 clinical samples was performed using the MGISEQ-200 platform (MGI Tech, Shenzhen, China) equipped with the pathogen fast identification system, following the manufacturer’s instructions. Additionally, 88 wastewater samples were sequenced using the BGI sequencing platform, as described in a previous study [12]. Briefly, wastewater sequencing on the BGI platform encompassed reverse transcription and amplification using an ATOPlex RNA multiplex PCR amplification kit V3.1, and a library preparation using MGIEasy fast PCR-FREE digestion library preparation kit. The DNBSEQ-G99 platform, which employed the single-end sequencing 100 bp + double-end barcode (SE100 + 10 + 10) strategy, was used for sequencing. Parametric assembly of SARS-CoV-2 sequences was conducted using the MGI MetargetCOVID software. For lineage abundance determination, MGI MetargetCOVID software was employed to perform quality control, alignment, and primer trimming. The Freyja tool (v1.3.12) was used to recover relative SARS-CoV-2 lineage abundances from wastewater samples based on the lineage-defining mutational ‘barcodes’ derived from the UShER global phylogenetic tree.

### 2.6. ddPCR Assays for XBB.1.16 and XBB.1.9

ddPCR assays for XBB.1.16 and XBB.1.9 were developed to assess variant abundance in wastewater samples. L3829F and G1819S were chosen to represent XBB.1.16 and XBB.1.9, respectively. The primers and probes used for detection and ddPCR are described in the Supplementary File S3.

### 2.7. Statistical Analysis

GraphPad Prism 8.0 software and Open-Source Epidemiologic Statistics for Public Health (OpenEpi, https://www.openepi.com/ accessed on 25 April 2023) were used for data analysis.

## 3. Results

### 3.1. MeltArray Assay Design

**Figure 1:**
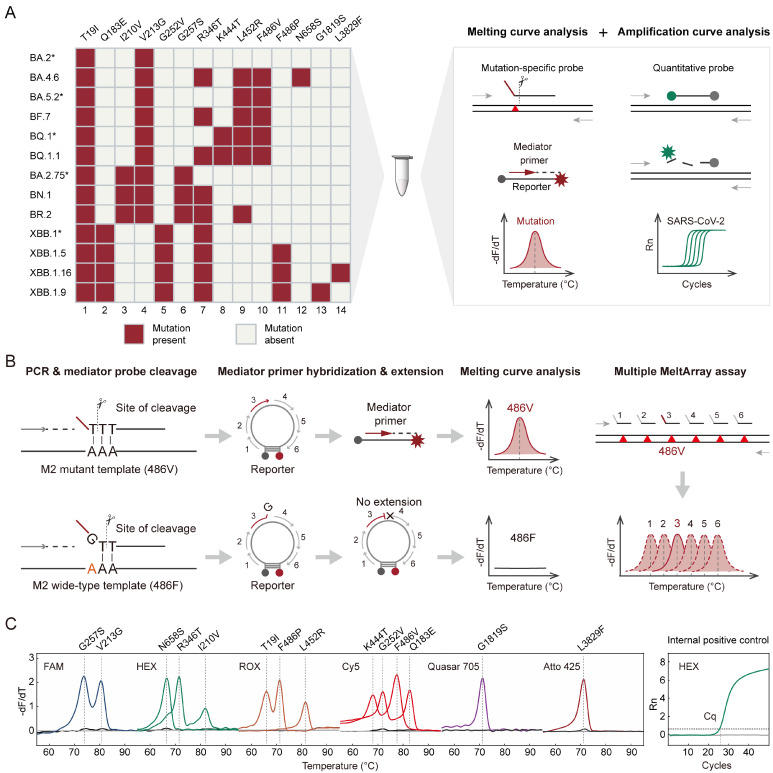
MeltArray assay for variant identification. (**A**) Signature mutations of the target Omicron variants (**left panel**) and their detection scheme (**right panel**). The notation “*” represents the lineage and its subvariants except those also listed. For mutation detection, mediator probes were designed to perfectly complement mutant templates, which can turn into a melting peak corresponding to the mutation. For viral quantification, a TaqMan probe was designed to target the conserved region of viral genome. The amplification curve also served as an internal positive control (IPC) for the entire assay. (**B**) The working principle of detecting single mutation via the MeltArray scheme. Taking the F486V mutation as an example, the mediator probe is perfectly matched to the mutant template, and the mediator primer cleaved via the Taq DNA polymerase can extend along the molecular beacon reporter and emit a fluorescent signal, which can turn into a melting peak corresponding with the mutation. In contrast, when the probe binds to the wild-type template, due to the mismatch of bases, an extra base “G” is cut off, and the mediator primer cannot extend along the reporter and fails to generate a fluorescent signal. The presence or absence of a melting peak thus indicates whether the site is wild-type or mutant. A molecular beacon reporter allows for the extension of multiple mediator primers to produce a series of fluorescent hybrids of different melting temperatures unique to each target. Using multiple molecular beacon reporters labeled with different fluorophores, the overall number of targets was equal to the number of the reporters multiplied by that of mediator primers per reporter. (**C**) Typical results of 14 variant-specific mutations and quantitative targets. Mutant templates are depicted by color curves, whereas wild-type templates are represented by black lines. Gray lines indicate the no-template control (NTC).

Omicron subvariants exhibited distinct mutation spectra, mostly in the spike gene. By comparing spike mutations among Omicron subvariants (mutation prevalence > 98%, data for 10 January 2023, www.outbreak.info), we identified 12 signature mutations (T19I, Q183E, I210V, V213G, G252V, G257S, R346T, K444T, L452R, F486V, F486P, and N658S) qualified to identify five major Omicron sublineages (BA.2*, BA.5.2*, BA.2.75*, BQ.1*, and XBB.1*), and six subvariants (BF.7, BN.1, BR.2, BQ.1.1, XBB.1.5, and BA.4.6) (Figure 1A). For XBB.1.16 and XBB.1.9, two ORF1ab mutations, L3829F and G1819S, were chosen because spike mutations alone were insufficient for identification. To detect these mutations in one reaction, we designed a multiplex RT-PCR assay according to the working principle of MeltArray [10] leveraging a site-specific cleavage strategy, as previously described [13]. Briefly, each mutation was labelled using a combination of fluorescence and melting peak temperatures (*T_m_*). The presence of a mutation was indicated by a corresponding melting peak induced via the mediator primer, which was specifically cleaved from the mediator probe (Figure 1B). In addition, as an internal positive control (IPC), the N gene of SARS-CoV-2 was detected using modified TaqMan chemistry, in which fluorescence was detected during the denaturation stage of PCR. The obtained Cq value was used to estimate the viral load.

The final MeltArray assay included real-time PCR detection of the N gene in one channel and a melting curve analysis of 14 mutations in six channels (Figure 1C). The detection results from the mutation-containing plasmids showed that both real-time PCR and melting curve analysis produced the expected results. The entire procedure was completed within 2.5 h after adding cDNA to the reaction.

### 3.2. Analytical Performance of MeltArray Assay

The analytical performance of the MeltArray assay was evaluated using cultured viral samples. The LOD experiments were performed at concentrations ranging from 5 to 50,000 copies per reaction. All variant-specific mutations were correctly detected in 20 replicates at concentrations ranging from 50 to 50,000 copies per reaction (Supplementary Figure S1). Linear relationships (R^2^ = 0.9978–0.9991) were obtained for Cq and logarithmic template concentrations. The LOD was determined to be 50 copies/reaction and the positive threshold was set at Cq ≤ 39. Tm reproducibility experiments revealed that 3 SDs of all *T_m_* values ranged from 0.16 °C to 0.30 °C (*n* = 24), the CV values were ≤0.14% (Table 1), and no cross-talk between adjacent melting peaks was observed. This study also showed the high stability of Cq values (3 SD = 0.46, CV = 0.43%). No false-positive signals were observed when tested with the wild-type template at concentrations up to 10^7^ copies/μL, demonstrating its high tolerance to the wild-type background (Figure 1C). When tested with nucleic acids extracted from 15 other respiratory viruses, no false-positive results were observed in either the melting curve or the amplification curve (Supplementary Table S2), indicating the high specificity of the MeltArray assay.

### 3.3. Clinical Evaluation of MeltArray Assay

**Figure 2:**
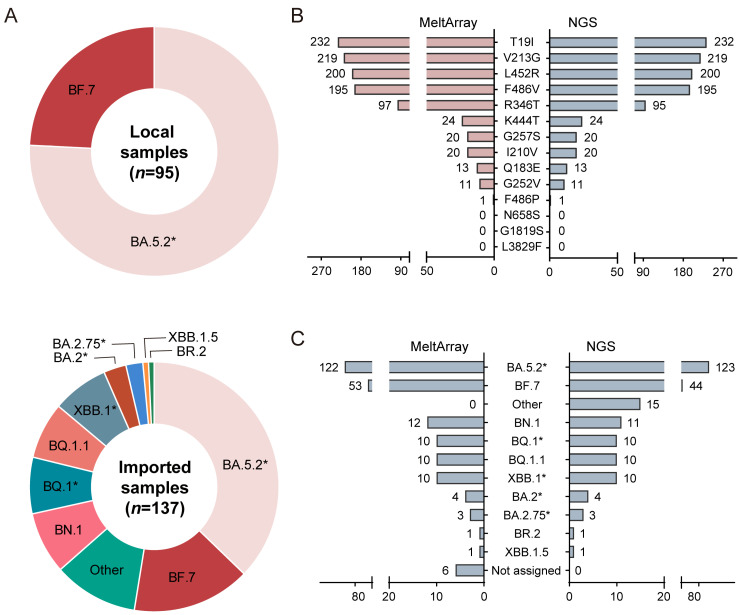
Clinical validation results. (**A**) Composition of SARS-CoV-2 variants from local and imported samples. The notation “*” represents the lineage and its subvariants except those also listed. (**B**) Comparison of the mutation detection results between the MeltArray assay and NGS in 232 SARS-CoV-2 samples. (**C**) Comparison of the variant identification results between the MeltArray assay and NGS in 232 SARS-CoV-2 samples.

We analyzed 239 SARS-CoV-2 positive samples using the MeltArray assay and compared the results with those obtained using NGS. Samples with Cq ≤ 39 (*n* = 232) were subjected to further analysis. The local samples (*n* = 95) mainly contained BA.5.2* and BF.7, whereas the imported samples (*n* = 137) comprised five sublineages and five subvariants, showing an increased diversity (Figure 2A). We first compared the quantitative results with those of a commercial RT-PCR test kit (targeting the ORF1ab and N genes) for SARS-CoV-2 nucleic acid detection. A high correlation between our assay and the commercial kit was observed in both the correlation (Supplementary Figure S2A,C,E) and Bland-Altman analyses (Supplementary Figure S2B,D,F).

We then compared the signature mutations detected via MeltArray with those detected via NGS. In total, nine signature mutations (T19I, V213G, L452R, F486V, R346T, K444T, G257S, I210V, Q183E, G252V, and F486P) were detected. All samples displayed identical signature mutation profiles using MeltArray and NGS (Figure 2B), except for two samples that displayed an additional minor R346T peak in MeltArray (Supplementary Figure S3A). Sanger sequencing confirmed the coexistence of 346T and 346R in the two samples (Supplementary Figure S3B), suggesting a mixed infection or carry-over contamination. With the omission of these situations, we concluded that the MeltArray assay correctly detected all signature mutations in the samples.

We further compared variant-typing consistency between MeltArray and NGS (Figure 2C). NGS generated five Omicron sublineages, BA.5.2*, BQ.1*, XBB.1*, BA.2*, and BA.2.75*, and five Omicron subvariants, BF.7, BN.1, BQ.1.1, BR.2, and XBB.1.5. Of the 232 samples, 213 (91.81%, 213/232) showed consistent variations between the two methods (Table 2). Of the 19 inconsistent samples, samples 1–6 were reported as unclassified variants via MeltArray and the other variants were outside the scope of MeltArray via NGS. Samples 7–19 showed inconsistent variants owing to additional mutations detected by NGS (Supplementary Table S3).

### 3.4. Local Epidemic Surveillance during the Omicron Outbreak

**Figure 3:**
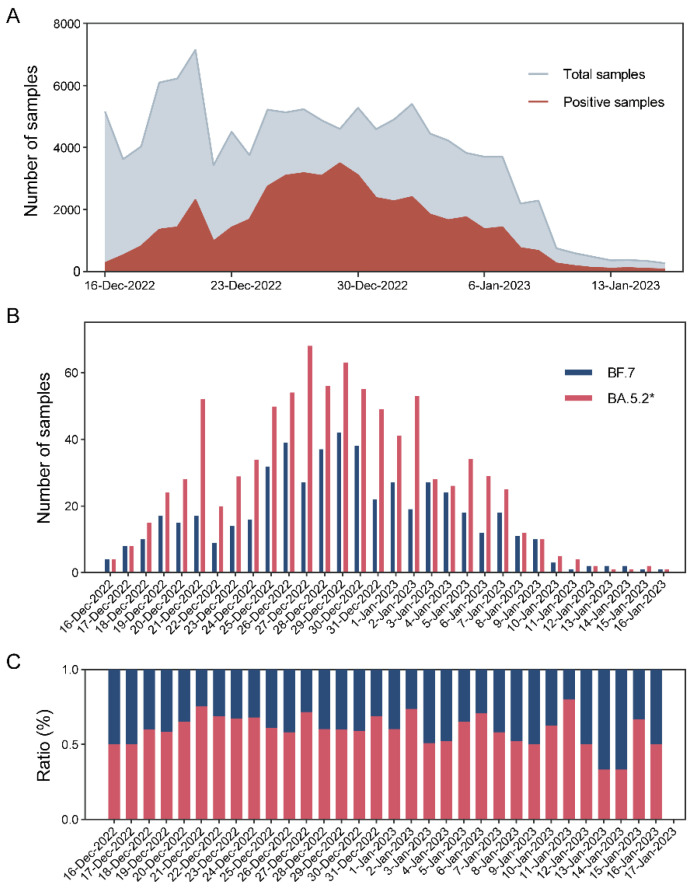
Temporal dynamics of local SARS-CoV-2 cases in Xiamen. (**A**) Total samples and SARS-CoV-2 positive cases detected by Zeesan medical laboratory. (**B**) Distribution of local subvariants over time. (**C**) Proportion of BF.7 and BA.5.2* in samples (*n* = 1408) assessed via the MeltArray assay.

A total of 116,700 samples were tested between 16 December 2022 and 16 January 2023 at the Zeesan Medical Laboratory. Among them, 46,873 (40.17%) were positive for COVID-19 (Figure 3A). A sampling rate of 3% was used each day, and 1408 samples were tested. The MeltArray assay results showed that all samples gave Cq ≤ 39, which ensured qualified variant identification. A total of 883 (62.71%) samples were identified as BA.5.2*, which harbored mutations in T19I, V213G, L452R, and F486V, whereas the remaining 525 (37.29%) samples were identified as BF.7, which harbored mutations in T19I, V213G, R346T, L452R, and F486V (Figure 3B). Daily statistics revealed that the entire outbreak was driven by BA.5.2* and BF.7, with a slightly higher frequency in the former than in the latter (Figure 3C).

### 3.5. Potential for Estimation of Variant Abundance via MeltArray Assay

**Figure 4:**
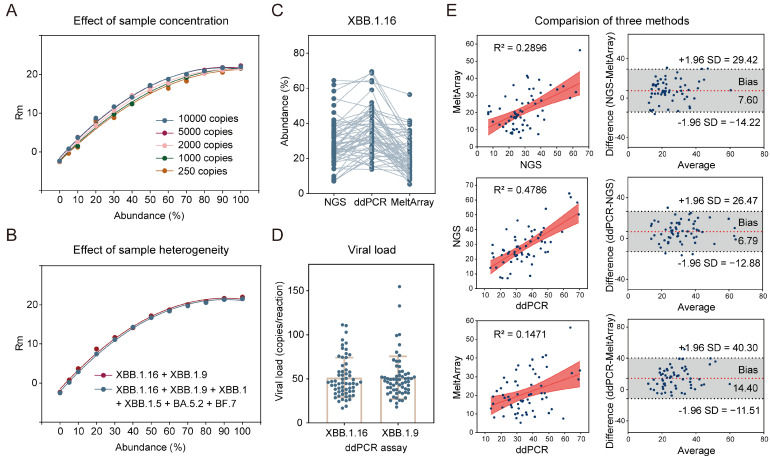
Abundance detection of XBB.1.16 in wastewater samples using MeltArray, NGS, and ddPCR. (**A**) Standard curves for Rm value and abundance detected using the MeltArray assay at different template concentrations. (**B**) Standard curves for Rm value and abundance detected using the MeltArray assay at different types or number of background variants with the total template concentration of 10,000 copies. (**C**) Comparison of the abundance in wastewater samples determined using NGS, ddPCR and MeltArray. (**D**) Viral load of XBB.1.16 and XBB.1.9 in wastewater samples detected using ddPCR assays. (**E**) Correlation and consistency analysis of the three methods. The left panel represents the Pearson correlation analysis, and the right panel represents the Bland–Altman analysis.

In the post-pandemic era, environmental surveillance is more cost effective than individual screening. However, wastewater samples frequently contain a mixture of viral lineages, mainly shed from infected individuals. Considering that the height of the melting peak (Rm) can indicate the relative abundance of the target, we explored the potential of the MeltArray assay to estimate variant abundance in co-infected samples. XBB.1.16 and XBB.1.9, which triggered a second wave of the Omicron epidemic in China starting in April 2023, were chosen for this investigation. The correlation between variant abundance and Rm value was studied using a series of mixtures containing different proportions of XBB.1.16 and XBB.1.9 (ranging from 100:0 to 5:95) at various template concentrations (10,000, 5000, 2000, 1000 and 250 copies/reaction). Each mixture was tested four times, and the average Rm values were calculated. A series of standard curves exhibited a nearly identical positive correlation between XBB.1.16 abundance and Rm values. Interestingly, the standard curves prepared from different template concentrations overlapped, indicating that this correlation was independent of the total viral concentration (Figure 4A). Similar results were obtained when replacing XBB.1.16 with XBB.1.9 (Figure S4A). When the coexisting variant was substituted with other variants or if the number of coexisting variants increased from one to five, identical results were obtained (Figure 4B), demonstrating the inherent stability of such correlations.

Taking advantage of the correlation established above, we tested wastewater samples collected during the second wave of the Omicron epidemic triggered by XBB.1.16 and XBB.1.9 between 8 May 2023 and 8 June 2023 in Shenzhen. Of the 88 samples analyzed, 62 exhibited Cq ≤ 39 and had mutation spectra of T19I, Q183E, G252V, R346T, F486P, G1819S, and L3829F, indicating the coexistence of XBB.1.16 and XBB.1.9. We also quantified their relative abundances based on the correlation established above and compared the results with those of ddPCR, the gold standard for nucleic acid quantification (Figure 4C). Surprisingly, variant abundances obtained from the two methods showed no significant correlations (Figure 4E and Figure S4C). Further comparison with NGS results, which are currently used for wastewater surveillance, also showed no correlation. We attributed these results to the extremely low viral load in the samples (51 copies/reaction on average), according to the ddPCR results (Figure 4D). These results highlighted the challenging nature of estimating variant abundance in wastewater samples at extremely low concentrations.

## 4. Discussion

Timely and continuous surveillance of emerging Omicron subvariants remains imperative given their persistent public health implications [14]. In this study, we developed a multiple RT-PCR assay using MeltArray. This assay enables the quantitative detection of SARS-CoV-2 and the rapid identification of Omicron subvariants. Because of the inherent nature of MeltArray, this assay can detect 14 mutations in one reaction, allowing the identification of five Omicron sublineages and eight subvariants based on their mutation spectra.

Several RT-PCR-based methods have been used for rapid screening of SARS-CoV-2 variants, including quantitative PCR (qPCR) [15,16] and high-resolution melting analysis (HRM) [17,18]. However, these methodologies were constrained by their ability to detect only three to four mutations in one reaction [19]. Mass spectrometry was used to detect multiple mutations in the SARS-CoV-2 variants. However, this method required multiple post-PCR manipulation steps, generally requiring 7–8 h [20,21], and its LOD varied greatly with mutations [22,23]. To overcome these limitations, we opted for the MeltArray scheme, which can theoretically accommodate over 60 targets within a single reaction via different combinations of fluorescent dyes and *T_m_* [10,13]. Previously, we established a single-reaction MeltArray assay to rapidly screen 32 representative mutations in Omicron BA.1. In this study, we integrated real-time PCR with melting curve analysis for simultaneous quantification and mutation identification in a single reaction. This design enabled us to infer the presence of SARS-CoV-2, estimate the relative viral load, and report the mutation profile of the virus without the need for pre-quantitative analysis.

The MeltArray assay operates on a real-time PCR thermal cycler, which is a widely available platform in molecular laboratories and enables the processing of 96 samples in one batch within 2.5 h. The simple procedure and automatic interpretation make it ideal for rapid mutation screening and extensive variant surveillance during local epidemics as seen in this study. We conducted a large-scale test on 1408 samples collected in Xiamen between 16 December 2022 and 16 January 2023 and revealed that the epidemic experienced in Xiamen after the adjustment of the control strategy in China was mainly dominated by BA.5.2* and BF.7. This result agrees with the observations of other studies conducted in China [24,25]. Interestingly, we noted the presence of multiple subvariants in the imported samples. However, they did not become dominant, presumably benefiting from the quarantine measures taken for imported cases at that time.

Wastewater-based surveillance (WBS) is a complementary surveillance method for estimating the prevalence of specific viral lineages, providing a community-wide snapshot of overall infection dynamics [26]. Similar to clinical testing, variant tracking in wastewater still relies on enrichment and sequencing of the environmental SARS-CoV-2 genome [27]. However, limited accessibility, high costs, and long turnaround impede the widespread and routine use of sequencing [28]. As an alternative strategy, PCR-based detection was proposed to provide real-time data for SARS-CoV-2 variants [29]. In this study, although the MeltArray assay correctly displayed all mutations in wastewater samples, variant abundances did not correlate well with NGS or ddPCR. In addition, a poor correlation was observed between NGS and ddPCR results. We attributed these results to the extremely low viral concentrations, which were unable to generate reproducible quantitative results regardless of the detection methods. Further studies are required to determine the cut-off viral loads above which the variants can be reliably quantified. Currently, our method may be better for inferring positivity rather than precisely quantifying the variant abundance in samples with low viral loads.

PCR-based methods provide limited genomic information compared to sequencing, thus, typed results were not as detailed as NGS results. However, our assay showed a high agreement with the NGS results (Table 2). Therefore, we assumed that as a detection technique for known mutations and variants, PCR-based methods could be used in combination with NGS, which mainly focuses on discovering emerging variants, whereas PCR methods such as MeltArray are used for routine monitoring [30]. Such a multitier monitoring framework should be more competitive than NGS alone in terms of cost-effectiveness, accessibility, and discriminatory power.

In conclusion, we have demonstrated the clinical utility of the MeltArray assay for variant surveillance and epidemic management. We anticipate that public health authorities could use the MeltArray assay to establish a network for real-time variant surveillance, wherein regular sampling and testing of different communities or key groups could be performed, allowing for the tracking of dynamic changes in prevalent SARS-CoV-2 variants. In this context, NGS can be used to periodically update systems to adapt to new variants. The combination of MeltArray and NGS could be a powerful monitoring tool that could be extensively used to handle infectious agents other than SARS-CoV-2.

## Figures and Tables

**Table 1:** Analytical performance of the MeltArray assay.

Gene Target	Codon Change	Channel	*T_m_* (℃)	CV (%)	Limit of Detection
			(Mean ± 3SD)		(Copies/Reaction)
T19I	ACA > ATA	ROX	66.02 ± 0.24	0.12	50
Q183E	CAG > GAG	Cy5	82.69 ± 0.21	0.08	50
I210V	ATT > GTT	HEX	81.76 ± 0.24	0.10	50
V213G	GTG > GGG	FAM	80.53 ± 0.23	0.10	50
G252V	GGT > GTT	Cy5	71.87 ± 0.22	0.10	50
G257S	GGT > AGT	FAM	73.40 ± 0.21	0.10	50
R346T	AGA > ACA	HEX	71.51 ± 0.22	0.10	50
K444T	AAG > ACG	Cy5	67.98 ± 0.16	0.08	50
L452R	CTG > CGG	ROX	81.55 ± 0.24	0.10	50
F486V	TTT > GTT	Cy5	77.63 ± 0.23	0.10	50
F486P	TTT > CCT	ROX	71.22 ± 0.24	0.11	50
N658S	AAC > AGC	HEX	66.71 ± 0.18	0.09	50
G1819S	GGT > AGT	Quasar 705	70.90 ± 0.23	0.11	50
L3829F	CTC > TTC	Atto 425	70.98 ± 0.30	0.14	50

**Table 2:** Comparison of MeltArray assay and NGS ^†^.

Variant	MeltArray Assay/NGS				Sensitivity% (95% CI)	Specificity% (95% CI)	PPV% (95% CI)	NPV% (95% CI)	Accuracy% (95% CI)	Kappa
	+/+	+/−	−/+	−/−						
BA.5.2*	117	5	6	104	95.12 (89.77–97.75)	95.41 (89.71–98.02)	95.90 (90.76–98.24)	94.55 (88.61–97.48)	95.26 (91.71–97.33)	0.90 (0.78–1.03)
BF.7	44	9	0	179	100 (91.97–100)	95.21 (91.15–97.46)	83.02 (70.77–90.80)	100 (97.90–100)	96.12 (92.79–97.95)	0.88 (0.76–1.01)
BN.1	11	1	0	220	100 (74.12–100)	99.55 (97.48–99.92)	91.67 (64.61–98.51)	100 (98.28–100)	99.57 (97.60–99.92)	0.95 (0.83–1.08)
BQ.1*	10	0	0	222	100 (72.25–100)	100 (98.30–100)	100 (72.25–100)	100 (98.30–100)	100 (98.37–100)	1 (0.87–1.13)
BQ.1.1	10	0	0	222	100 (72.25–100)	100 (98.30–100)	100 (72.25–100)	100 (98.30–100)	100 (98.37–100)	1 (0.87–1.13)
XBB.1*	10	0	0	222	100 (72.25–100)	100 (98.30–100)	100 (72.25–100)	100 (98.30–100)	100 (98.37–100)	1 (0.87–1.13)
BA.2*	4	0	0	228	100 (51.01–100)	100 (98.34–100)	100 (51.01–100)	100 (98.34–100)	100 (98.37–100)	1 (0.87–1.13)
BA.2.75*	3	0	0	229	100 (43.85–100)	100 (98.35–100)	100 (43.85–100)	100 (98.35–100)	100 (98.37–100)	1 (0.87–1.13)
XBB.1.5	1	0	0	231	100 (20.65–100)	100 (98.36–100)	100 (20.65–100)	100 (98.36–100)	100 (98.37–100)	1 (0.87–1.13)
BR.2	1	0	0	231	100 (20.65–100)	100 (98.36–100)	100 (20.65–100)	100 (98.36–100)	100 (98.37–100)	1 (0.87–1.13)

^†^ Data were analyzed using Open Source Epidemiologic Statistics for Public Health (OpenEpi, https://www.openepi.com/ (accessed on 25 April 2023)) and reported as estimate (95% CI). The notation “*” represents the lineage and its subvariants except those also listed.

## Data Availability

Data are contained within the article and Supplementary Materials.

## Supplementary Materials

The following supporting information can be downloaded at: https://www.mdpi.com/article/10.3390/v15122397/s1. File S1. ddPCR assay for culture virus quantification; File S2. RNA extraction and semiquantitative RT-PCR of samples; File S3. Component and procedure of the ddPCR assay for XBB.1.16 and XBB.1.9; Table S1. Primers and probes used in the MeltArray assay; Table S2. Detection results of other respiratory viruses by MeltArray assay; Table S3. Inconsistent variant-typing results between MeltArray assay and NGS; Figure S1. The limit of detection (LOD) of MeltArray assay; Figure S2. Correlation analysis of viral loads obtained from the MeltArray assay and commercial kit targeting ORF1ab and N gene; Figure S3. Sanger sequencing validation of inconsistent samples; Figure S4. Abundance detection of XBB.1.9 using MeltArray, NGS, and ddPCR.
