# Supplementary material for: Epidemiological Insights into the Omicron Outbreak via MeltArray-Assisted Real-Time Tracking of SARS-CoV-2 Variants
Source: Viruses. 2023 Dec 8;15(12):2397. doi: 10.3390/v15122397 (PMC10748191; doi:10.3390/v15122397)
Supplement: Supplementary file 1 [file viruses-15-02397-s001.zip › viruses-2724349-supplementary.pdf]

File S1. ddPCR assay for culture virus quantification.

ddPCR was performed using the TD1 Droplet Digital PCR System (Targeting One, Beijing, China) according to the manufacturer's instructions. A 30-μL ddPCR mix was prepared with 15 μL of 2 × Probe ddPCR SuperMix (TargetingOne, Beijing, China), 800 nM primers (forward, 5′-GCTGCTGCTTGACAGATTGA-3′ and reverse, 5′-GCCTCAGCAG-CAGATTTCTT-3′), 250 nM probes (5′-HEX- ACAGTTTGGCCTTGTTGTTGTTGGCC-BHQ1-3′), and 5 μL DNA template.

File S2. RNA extraction and semiquantitative RT-PCR of samples.

Three sample batches were used in the clinical study. The first batch of 239 SARS-CoV-2 RNA samples was extracted using the GeneRotex96 Nucleic Acid Extractor and accompanying kit (Tianlong Science and Technology, Xi'an, China) and screened using a semi-quantitative RT-PCR kit (Triplex International Biosciences, Xiamen, China) with amplification targeting the ORF1ab and N genes. The second batch comprised 1408 SARS-CoV-2 RNA samples was extracted using the Lab-Aid 960 Nucleic Acid Extractor and accompanying kit (Zeesan Biotech, Xiamen, China) and screened using a semiquantitative RT-PCR kit (EasyDiagnosis Biomedicine, Wuhan, China) with amplification targeting the ORF1ab and N genes. The third batch comprised 88 SARS-CoV-2 RNA samples from wastewater was extracted using automatic nucleic acid extraction platform HBNP-9601A (Hybribio, Guangdong, China) and screened using a semiquantitative RT-PCR kit (Hybribio, Guangdong, China) with amplification targeting the ORF1ab and N genes.

File S3. Component and procedure of the ddPCR assay for XBB.1.16 and XBB.1.9.

ddPCR was performed using the TD1 Droplet Digital PCR System (Targeting One, Beijing, China). A 30-μL dPCR mix was prepared with 15 μL of 2 × Probe ddPCR SuperMix (TargetingOne, Beijing, China), 800 nM forward primers (L3829F:5′-AACCGCTACTTTA-GACTGACTCTT-3′)(G1819S:5′-AAAATATCTAGTACAACAGGAGTCACC-3′) and reverse primers (L3829F:5′-AGGTTTGCCACCAACACC-3′)(G1819S:5′-GTAAAGCAC-CGTCTATGCAATACA-3′), 250 nM probes (L3829F:5′-FAM-AGGGACTACTCCCACC-BHQ1-3′ and 5′-HEX-AGGGACTATTCCCACCC-BHQ1-3′) (G1819S:5′-FAM-CTTAA-GCATGGTACATTTAC-BHQ1-3′ and 5′-HEX-CTTAAGCATAGTACATTTACTTGT-BHQ1-3′) (Note: The underlined nucleotides indicate locked nucleic acids) and 5 μL DNA template. After droplet generation, polymerase chain reaction (PCR) was performed using an A300 Fast Thermal Cycler (Longgene, Hangzhou, China). The following thermal cycling program was deployed: denaturation at 95 °C for 10 min, followed by 40 cycles of 94 °C for 30 s and 58 °C for 1 min (ramp rate of 1.5 °C per second), and maintenance at 12 °C. The endpoint fluorescence of each droplet was measured using the TD1 droplet reader (Targeting One). ChipReader R1 software was used for the quality control and preliminary analysis of the raw data. The overall viral load in each sample was calculated using all positive droplets. The mutant fraction (MF), as exemplified by the HEX-labelled probe, was calculated using the following equation: HEX copies / (FAM copies + HEX copies), where HEX represents mutant droplets and FAM represents wild-type droplets.

Table S1. Primers and probes used in the MeltArray assay.

Oligo	Sequence (5′ → 3′) †	Concentration (nM)	Length (nt)
F1 (*S*: c.2)	GCAAGCCCTCACGTAGCGAATGTTTGTTTTTCTTGTTTTATTGC	40	44
R1 (*S*: c.313)	GCAAGCCCTCACGTAGCGAAATCCAGCCTCTTATTATGTTAGA	40	43
T19I-P	CACCTCTCACATAAGATTAACACA$\underline{C}$TGACTAGAGACTAGTG-C_7NH_2	40	41
F2 (*S*: c.518)	GCAAGCCCTCACGTAGCGAAAGCCTTTTCTTATGGACCTT	40	40
R2 (*S*: c.869)	GCAAGCCCTCACGTAGCGAATCTACAGCATCTGTAATGGTT	40	41
Q183E-P	CTGCTCCTCCTGAGGGTAATTT$\underline{C}$AAAAATCTTAGGGAATTTGTGTT-C_7NH_2	200	46
I210V-P	AGACGAGCCGTGTCAGGCGTGTGCTTAGAATATATTTAAAATAACCA-C_7NH_2	400	48
V213G-P	CCGTGCTCACCTGGCGTGATCTCCCTCAGGCACGG-C_7NH_2	200	35
G252V-P	TGACTTCCACACAGGAGTCAAATAACTTCTATGTAAAGCAAGTAA-C_7NH_2	200	45
G257S-P	CCTGTGGTCGTTGAAGAAGAATCAMCAGGAGTCAAATAACT-C_7NH_2	400	41
F3 (*S*: c.903)	GCAAGCCCTCACGTAGCGAATACGTTGAAATCCTTCACTG	40	40
R3 (*S*: c.1192)	GCAAGCCCTCACGTAGCGAACTGCATAGACATTAGTAAAGCA	40	42
R346T-P	CTCTGACTCTCATTTGCATCTGTTTATGCTTGGAACAGGAA-C_7NH_2	200	41
F4 (*S*: c.1229)	GCAAGCCCTCACGTAGCGAATCGCTCCAGGGCAAA	40	35
R4 (*S*: c.1541)	GCAAGCCCTCACGTAGCGAAGAAAGTACTACTACTCTGTATGGT	40	44
K444T-P	TCCACATCGTTGTA$\underline{G}$AATCAAGCTTGTTAGAATTCCAAGCT-C_7NH_2	400	41
L452R-P	ACACTGTCCAGGTATAGATTGTTTAGGAAGTCTAATCTCAAACCTT-C_7NH_2	400	46
F486V-P	TGCCACGACGTTAATT$\underline{G}$TTACTTTCCTTTACAATCATATGGTTTCC-C_7NH_2	400	46
F486P-P	TCATCACACCCTAATTGTTACTCTCCTTTACAATCATATGGTTTCC-C_7NH_2	20	46
F5 (*S*: c.1909)	GCAAGCCCTCACGTAGCGAATCTACAGGTTCTAATGTTTTTCA	40	43
R5 (*S*: c.2102)	GCAAGCCCTCACGTAGCGAAGCACCAAGTGACATAGTGTA	40	40
N658S-P	CTCTCTCTGGCTCATATGAGTGTGACATACCCATTGGT-C_7NH_2	200	38
F6 (*ORF1a*: c.5400)	GCAAGCCCTCACGTAGCGAAGGAGTCACCTTTTGTTATGAT	80	41
R6 (*ORF1a*: c.5638)	GCAAGCCCTCACGTAGCGAACTGGTTTTATGGTTGTTGTGTA	80	42
G1819S-P	CGTGTGACTGAGTACATTTACTTGTGCTAGTGAGTACACTGG-C_7NH_2	400	42
F7 (*ORF1a*: c.11310)	GCAAGCCCTCACGTAGCGAACATAACTGGTAATACACTTCAGT	80	43
R7 (*ORF1a*: c.11603)	GCAAGCCCTCACGTAGCGAAGTGCACTTTACATCTGACATT	80	41
L3829F-P	CGGTACTCTCTTCCCACCCAAGAATAGCATAGATGTACCG-C_7NH_2	400	40
F8 (*N*: c.622)	GCAAGCCCTCACGTAGCGAAGCTAGAATGGCTGGCAA	20	37
R8 (*N*: c.805)	GCAAGCCCTCACGTAGCGAATGTATGCTTTAGTGGCAGTA	20	40
IPC-P	HEX-ACAGTTTGGCCTTGTTGTTGTTGGCC-BHQ1	100	26
Universal primer	GCAAGCCCTCACGTAGCGAA	800	20

†Underlined nucleotides indicate locked nucleic acids.

Table S2. Detection results of other respiratory viruses by MeltArray assay†.

Num.	Strains	T19I	Q183E	I210V	V213G	G252V	G257S	R346T	K444T	L452R	F486V	F486P	N658S	G1819S	L3829F	IPC
1	CoV-OC43	ND	ND	ND	ND	ND	ND	ND	ND	ND	ND	ND	ND	ND	ND	ND
2	CoV-NL63	ND	ND	ND	ND	ND	ND	ND	ND	ND	ND	ND	ND	ND	ND	ND
3	CoV-OC229E	ND	ND	ND	ND	ND	ND	ND	ND	ND	ND	ND	ND	ND	ND	ND
4	InfA-H3N2	ND	ND	ND	ND	ND	ND	ND	ND	ND	ND	ND	ND	ND	ND	ND
5	InfB-Y	ND	ND	ND	ND	ND	ND	ND	ND	ND	ND	ND	ND	ND	ND	ND
6	InfB-V	ND	ND	ND	ND	ND	ND	ND	ND	ND	ND	ND	ND	ND	ND	ND
7	HPIV-1	ND	ND	ND	ND	ND	ND	ND	ND	ND	ND	ND	ND	ND	ND	ND
8	HPIV-3	ND	ND	ND	ND	ND	ND	ND	ND	ND	ND	ND	ND	ND	ND	ND
9	HRV-B52	ND	ND	ND	ND	ND	ND	ND	ND	ND	ND	ND	ND	ND	ND	ND
10	HAdV	ND	ND	ND	ND	ND	ND	ND	ND	ND	ND	ND	ND	ND	ND	ND
11	RSVA	ND	ND	ND	ND	ND	ND	ND	ND	ND	ND	ND	ND	ND	ND	ND
12	RSVB	ND	ND	ND	ND	ND	ND	ND	ND	ND	ND	ND	ND	ND	ND	ND
13	HMPV-A2	ND	ND	ND	ND	ND	ND	ND	ND	ND	ND	ND	ND	ND	ND	ND
14	HBOV	ND	ND	ND	ND	ND	ND	ND	ND	ND	ND	ND	ND	ND	ND	ND
15	MP	ND	ND	ND	ND	ND	ND	ND	ND	ND	ND	ND	ND	ND	ND	ND

†ND: not detected; IPC: internal positive control.

Table S3. Inconsistent variant-typing results between MeltArray assay and NGS.

Num.	NGS	MeltArray	Mutation detected	Differential region	Specific mutation
1	Other (BM.4.1.1: BA.2.75.3.4.1.1)	Not assigned	Consistent	/	/
2	Other (BM.4.1.1: BA.2.75.3.4.1.1)	Not assigned	Consistent	/	/
3	Other (BM.4.1.1: BA.2.75.3.4.1.1)	Not assigned	Consistent	/	/
4	Other (BM.4.1.1: BA.2.75.3.4.1.1)	Not assigned	Consistent	/	/
5	Other (XBB.2.2)	Not assigned	Consistent	/	/
6	Other (XBB.3)	Not assigned	Consistent	/	/
7	Other (BA.5.1.10)	BA.5.2*	Consistent	*ORF1a;* *ORF1b;* *ORF10;*	*ORF1a*: K3839R; *ORF1b*: T1050N; *ORF10*: L37F;
8	Other (BF.27: BA.5.2.1.27)	BA.5.2*	Consistent	*ORF1a;* *ORF1b;* *ORF8;*	*ORF1a*: A2784V; *ORF1b*: T1050N; *ORF8*: A65D;
9	Other (BF.11: BA.5.2.1.11)	BF.7 (BA.5.2.1.7)	Consistent	*N*	*N*: Del30/32; Del31/33; S33F
10	Other (BE.1: BA.5.3.1.1)	BA.5.2*	Consistent	*ORF1a;* *ORF1b;* *N;*	*ORF1a*: Q556K; *ORF1b*: T1050N; M1156I; *N*: E136D

11	Other (BA.5.1)	BA.5.2*	Consistent	*ORF1b;*<br>*ORF10;*	*ORF1b*: T1050N;<br>*ORF10*: L37F;
12	Other (BA.5.5.1)	BA.5.2*	Consistent	*ORF1b;*<br>*S;*<br>*ORF8;*	*ORF1b*: T1050N;<br>*S*: T76I; N450D;<br>*ORF8*: V62L;
13	Other<br>(CR.1:<br>BA.5.2.18.1)	BF.7<br>(BA.5.2.1.7)	Consistent	*ORF1b;*<br>*S;*<br>*N;*	*ORF1b*: T1050N;<br>*S*: Del144; K444R;<br>*N*: Del30/32; Del31/33; S33F
14	Other<br>(BL.2:<br>BA.2.75.1.2)	BN.1<br>(BA.2.75.5.1)	Consistent	*S*	*S*: K356T; F490S; D574V;
15	Other (DQ.1)	BF.7<br>(BA.5.2.1.7)	Consistent	*ORF1b;*<br>*S;*<br>*N;*	*ORF1b*: T1050N;<br>*S*: K147N;<br>*N*: Del30/32; Del31/33; S33F
16	BA.5.2*<br>(BA.5.2.6)	BF.7<br>(BA.5.2.1.7)	Consistent	*ORF1b;*<br>*N;*	*ORF1b*: T1050N;<br>*N*: Del30/32; Del31/33; S33F
17	BA.5.2*<br>(BA.5.2.47)	BF.7<br>(BA.5.2.1.7)	Consistent	*ORF1b;*<br>*N;*	*ORF1b*: T1050N;<br>*N*: Del30/32; Del31/33; S33F
18	BA.5.2*<br>(BA.5.2.47)	BF.7<br>(BA.5.2.1.7)	Consistent	*ORF1b;*<br>*N;*	*ORF1b*: T1050N;<br>*N*: Del30/32; Del31/33; S33F
19	BA.5.2*<br>(BA.5.2.1)	BF.7<br>(BA.5.2.1.7)	Consistent	*S*	*S*: R346T;

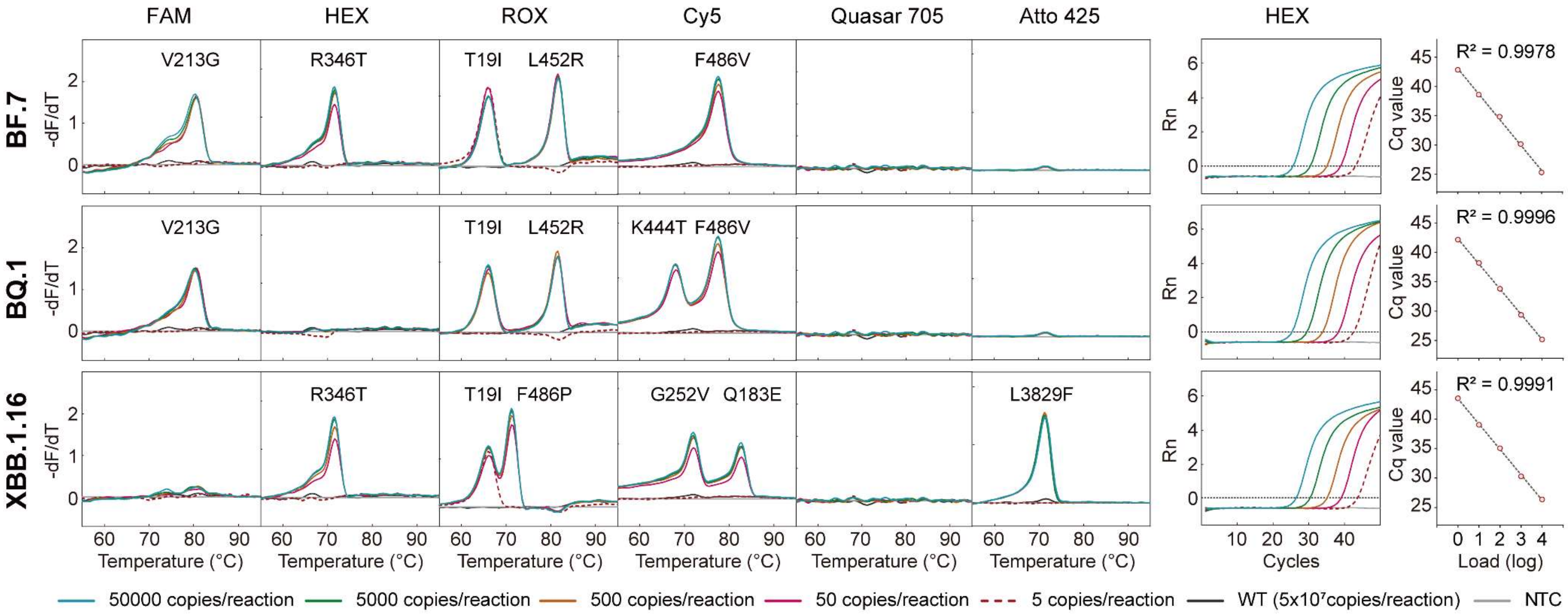

Figure S1. The limit of detection (LOD) of MeltArray assay. Ten-fold serial dilutions of the Omicron samples ranging from 5 copies/reaction to 50000 copies/reaction were detected. Black lines indicate wild-type, and gray lines indicate no-template control (NTC).

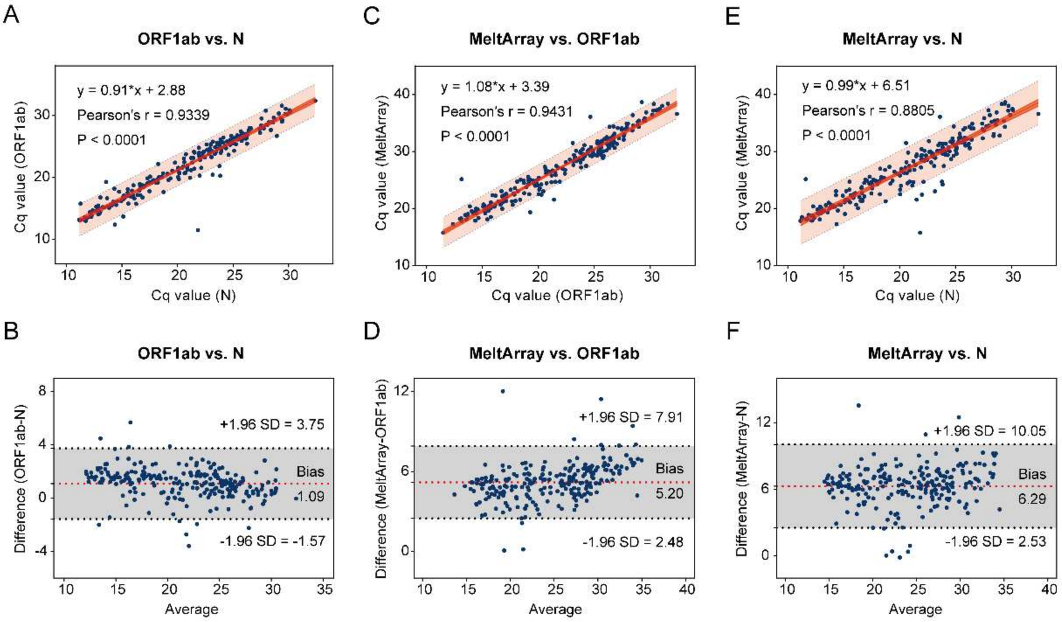

Figure S2. Correlation analysis of viral loads obtained from the MeltArray assay and commercial kit targeting ORF1ab and N gene. (ACE) Pearson correlation analysis. The dark red shade represents 95% confidence band, the light red shade represents 95% prediction band. (BDF) Bland–Altman analysis. The grey shaded area is within 1.96 times standard deviation. The red dot line represents the averaged ratio, which is given for each panel. The consistency of the viral loads obtained from the three targets in detecting 232 SARS-CoV-2 samples was shown by the correlation coefficients (0.8805-0.9431) of Pearson correlation analysis and the difference (1.09-6.29) of Bland–Altman analysis.

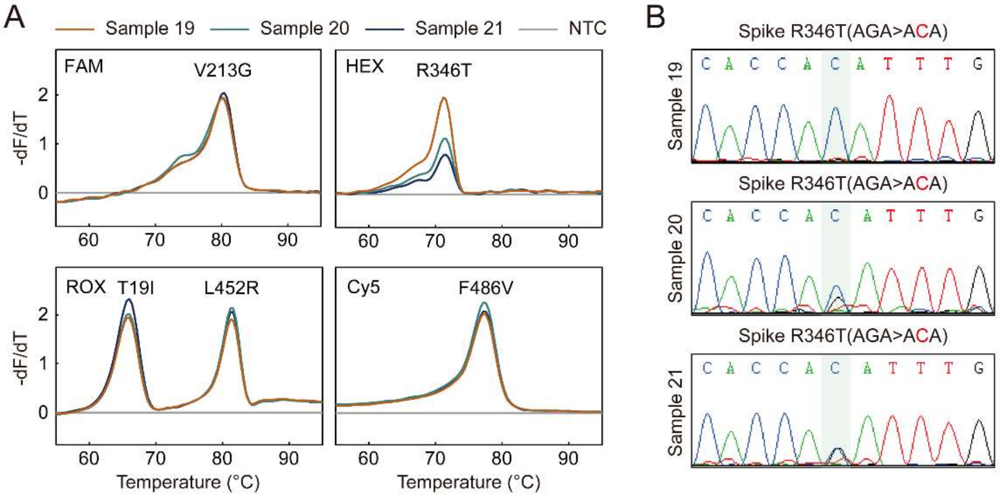

Figure S3. Sanger sequencing validation of inconsistent samples. (**A**) The results obtained by the MeltArray assay. R346T mutation is only detected by MeltArray assay in sample 20 and 21. Sample 19 was taken as a control, in which the R346T mutation was detected by both NGS and MeltArray. (**B**) The results confirmed by Sanger sequencing.

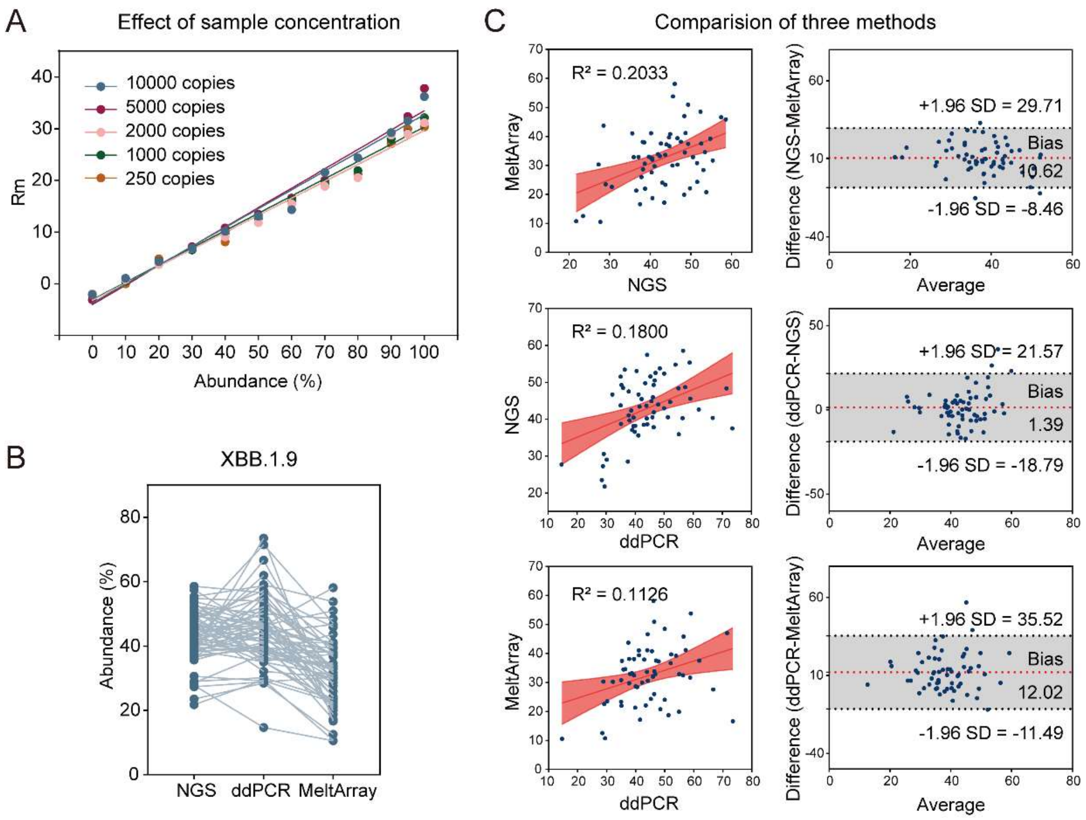

Figure S4. Abundance detection of XBB.1.9 using MeltArray, NGS, and ddPCR. (**A**) Working curves for Rm value and abundance detected by the MeltArray assay under different template concentration. (**B**) Abundance of wastewater samples reported by three methods. (**C**) The correlation and consistency analysis of three methods.
